# Supplementary material for: Interventions to increase help-seeking for mental health care in low- and middle-income countries: A systematic review
Source: PLOS Glob Public Health. 2023 Sep 13;3(9):e0002302. doi: 10.1371/journal.pgph.0002302 (PMC10499262; doi:10.1371/journal.pgph.0002302)
Supplement: S1 Table — (DOCX) [file pgph.0002302.s001.docx]

**S1 Table. PRISMA checklist**

| **Section and Topic** | **Item #** | **Checklist item** | **Location where item is reported** | **Evidence from manuscript** |
| --- | --- | --- | --- | --- |
| **TITLE** | | |  |  |
| Title | 1 | Identify the report as a systematic review. | P. 1 | Interventions to Increase Help-seeking for Mental Health Care in Low-and-Middle-Income Countries: A Systematic Review |
| **ABSTRACT** | | |  |  |
| Abstract | 2 | See the PRISMA 2020 for Abstracts checklist. | P. 1, 2 | Mental health problems are a significant and growing cause of morbidity worldwide. Despite the availability of evidence-based interventions, most people experiencing mental health problems remain untreated. This treatment gap is particularly large in in low- and middle-income countries (LMIC) and is due to both supply-side and demand-side barriers. The aim of this systematic review is to identify and synthesise the evidence on interventions to improve help-seeking for mental health problems in LMICs. The protocol was registered a priori (Registration number: CRD42021255635). We searched eight databases using terms based on three concepts: ‘mental health/illness’ AND ‘help-seeking’ AND ‘LMICs’; and included all age groups and mental health problems. Forty-two papers were eligible and included in this review. Intervention components were grouped into three categories following the steps in the help-seeking process: (1) raising mental health awareness among the general population (e.g., distribution of printed or audio-visual materials), (2) identification of individuals experiencing mental health problems (e.g., community-level screening or detection), and (3) promoting help-seeking among people in need of mental health care (e.g., sending reminders). The majority of interventions (80%) included components in a combination of the aforementioned categories. Most studies report positive outcomes, yet results on the effectiveness is mixed, with a clear trend in favour of interventions with components from more than one category. Ten out of 42 studies (24%) yielded a statistically significant effect of the intervention on help-seeking; and all targeted a combination of the aforementioned categories (i.e., raising awareness, identification and help-seeking promotion). Only six studies (14%) focused on children and adolescents. Due to the limited number of robust studies done in LMICs and the heterogeneity of study designs, outcomes and components used, no definite conclusions can be drawn with regards to the effects of individual strategies or content of the interventions. |
| **INTRODUCTION** | | |  |  |
| Rationale | 3 | Describe the rationale for the review in the context of existing knowledge. | P. 3 | In addition, both previously conducted reviews only included trials, and none of these studies were conducted in low-income countries. Only three studies were based in the same middle-income country, China [20]. Considering that mental health care utilisation remains particularly low in LMICs [4] and that barriers contributing to this gap are context-driven and especially prominent in such settings, it is vital to identify contextual strategies to improve help-seeking. To build on the findings of the previously conducted reviews, this review included study designs beyond trials and also included regional databases. |
| Objectives | 4 | Provide an explicit statement of the objective(s) or question(s) the review addresses. | P. 4 | The aim of this systematic review is to identify and synthesise the evidence on interventions to improve help-seeking for mental health problems in LMICs.  The specific objectives of this review are to: (1) Synthesise the effectiveness of interventions in improving help-seeking behaviour, service utilisation and contact coverage; (2) Describe and categorise components and implementation processes of the interventions; (3) Synthesise information on factors that mediate help-seeking; and (4) Synthesise information about stakeholder perceptions and implementation-related outcomes of the interventions. |
| **METHODS** | | |  |  |
| Eligibility criteria | 5 | Specify the inclusion and exclusion criteria for the review and how studies were grouped for the syntheses. | P. 5, 7 | Eligibility criteria were: (i) studies conducted in a LMIC, according to the World Bank categorisation [25]; (ii) peer-reviewed papers published in English; (iii) study participants of all ages, to distinguish it from previous reviews that focussed solely on children or adults; (iv) presentation of primary data; (v) evaluation of an intervention with a stated aim of promoting help-seeking behaviour; and (vi) reporting on at least one help-seeking behaviour related outcome (e.g., service utilisation or contact coverage). Studies evaluating the same intervention but conducted in another context or focusing on different outcomes were included as separate studies. Experimental, quasi-experimental and non-experimental studies were included.  Considering the heterogeneity in study designs and outcome measures, results were reported descriptively as a narrative synthesis [31]. Sub-groups were created based on study design, outcome measures and intervention content (i.e., intervention components). Intervention components were grouped into three categories following a process model of help-seeking that conceptualises help-seeking as a dynamic multi-stepped process [23]. |
| Information sources | 6 | Specify all databases, registers, websites, organisations, reference lists and other sources searched or consulted to identify studies. Specify the date when each source was last searched or consulted. | P. 4, 5 | We searched eight databases between the 24th and 27th of May 2021, no publication date range were set in the databases: Medline, PsycINFO, Embase, Global Health, Cochrane Central, Latin American and Caribbean Health Sciences Literature (LILACS), Scientific Electronic Library Online (SCiELO), and African Journals Online (AJOL). We also searched for potentially eligible studies in the reference lists of the two aforementioned systematic reviews [19,20]. Additionally, we identified studies through forward and backward reference searching in Web of Science. Finally, four independent experts who are involved in help-seeking-related research in LMICs were contacted to identify additional published literature. We updated our results by re-running the search again between 9th and 16th January 2023 to account for studies that were published after May 2021 (when the search was first run). |
| Search strategy | 7 | Present the full search strategies for all databases, registers and websites, including any filters and limits used. | S4 | Attached as S4: Search strategy (Medline) |
| Selection process | 8 | Specify the methods used to decide whether a study met the inclusion criteria of the review, including how many reviewers screened each record and each report retrieved, whether they worked independently, and if applicable, details of automation tools used in the process. | P. 5 | After conducting the search, the results were imported to Endnote for automatic and manual de-duplication. The results were then uploaded to Covidence for title and abstract screening. Two researchers (MvdB and YG) independently screened the abstracts (n=23,215); 8% were screened by both. In cases of disagreement, a third researcher (DSS) independently assessed eligibility. The remaining abstracts were screened by one of the researchers (MvdB and YG). Subsequently, each potentially eligible full-text article was independently assessed by two out of four researchers (MvdB, YG, DSS, or VeP). In cases where consensus was not reached, additional researchers (AN and MJ) determined eligibility. In the rare cases where the full version of the publication could not be found online, the corresponding author was contacted via email, and it was excluded if they did not respond within two weeks. |
| Data collection process | 9 | Specify the methods used to collect data from reports, including how many reviewers collected data from each report, whether they worked independently, any processes for obtaining or confirming data from study investigators, and if applicable, details of automation tools used in the process. | P. 6 | Two researchers (MvdB and YG) piloted the data extraction sheet by independently extracting information from 10% of the eligible papers. Data from the remaining papers were extracted by one of the researchers and reviewed by the other researcher. Some papers did not provide sufficient intervention details, and thus we accessed the paper the authors referenced for the intervention description. However, this was not included in our final list of included papers as it did not report on any relevant outcomes. |
| Data items | 10a | List and define all outcomes for which data were sought. Specify whether all results that were compatible with each outcome domain in each study were sought (e.g. for all measures, time points, analyses), and if not, the methods used to decide which results to collect. | P. 6 | The primary outcomes of interest were:   1. Help-seeking behaviour: any action taken to seek formal or informal help from mental healthcare services, or trusted individuals in the community [16]. 2. Service utilisation: visiting any type of formal mental healthcare services [26]. 3. Contact coverage: proportion of individuals in need of mental health treatment who seek help [8,27]. 4. Help-seeking efficacy: perceived helpfulness in promoting help-seeking [28].   Secondary outcomes of interest included hypothesised or studied mediators affecting help-seeking, implementation outcomes as defined by Proctor et al. [29] and, process indicators such as quality of training, session attendance, etc. |
|  | 10b | List and define all other variables for which data were sought (e.g. participant and intervention characteristics, funding sources). Describe any assumptions made about any missing or unclear information. | P. 6 | Data was extracted on five key domains: general target study areas (country, mental health conditions, target population, setting); intervention (description, specific components, mode and agent of delivery, duration, frequency of sessions); primary outcome measures and results; hypothesised/studied mediators; and implementation-related outcomes. |
| Study risk of bias assessment | 11 | Specify the methods used to assess risk of bias in the included studies, including details of the tool(s) used, how many reviewers assessed each study and whether they worked independently, and if applicable, details of automation tools used in the process. | P. 7 | The risk of bias was assessed by two reviewers (MvdB and YG) using the Joanna Briggs Institute (JBI) critical appraisal tools relevant for the appropriate study design [30]. It was used to evaluate the methodological quality and the possibility of bias in its design, conduct and analysis. After independent evaluation, the two reviewers met to reach consensus on the score. The quality of each study did not impact the weight given to it in the narrative synthesis. |
| Effect measures | 12 | Specify for each outcome the effect measure(s) (e.g. risk ratio, mean difference) used in the synthesis or presentation of results. | P. 7 | Considering the heterogeneity in study designs and outcome measures, results were reported descriptively as a narrative synthesis [31]. Implementation outcomes were presented based on common themes. |
| Synthesis methods | 13a | Describe the processes used to decide which studies were eligible for each synthesis (e.g. tabulating the study intervention characteristics and comparing against the planned groups for each synthesis (item #5)). | P. 7 | Sub-groups were created based on study design, outcome measures and intervention content (i.e., intervention components). Intervention components were grouped into three categories following a process model of help-seeking that conceptualises help-seeking as a dynamic multi-stepped process [23]. This was summarised in a series of tables and graphical representations. Findings relating to effectiveness were summarised based on the intervention content. |
|  | 13b | Describe any methods required to prepare the data for presentation or synthesis, such as handling of missing summary statistics, or data conversions. | P. 7 | Intervention components were grouped into three categories following a process model of help-seeking that conceptualises help-seeking as a dynamic multi-stepped process [23]. This was summarised in a series of tables and graphical representations. Findings relating to effectiveness were summarised based on the intervention content. |
|  | 13c | Describe any methods used to tabulate or visually display results of individual studies and syntheses. | P, 8; Table 1 | Attached as Table 1: Characteristics of included studies and interventions |
|  | 13d | Describe any methods used to synthesize results and provide a rationale for the choice(s). If meta-analysis was performed, describe the model(s), method(s) to identify the presence and extent of statistical heterogeneity, and software package(s) used. | P. 7 | Considering the heterogeneity in study designs and outcome measures, results were reported descriptively as a narrative synthesis [31]. |
|  | 13e | Describe any methods used to explore possible causes of heterogeneity among study results (e.g. subgroup analysis, meta-regression). | P. 7 | Considering the heterogeneity in study designs and outcome measures, results were reported descriptively as a narrative synthesis [31]. Sub-groups were created based on study design, outcome measures and intervention content (i.e., intervention components). Intervention components were grouped into three categories following a process model of help-seeking that conceptualises help-seeking as a dynamic multi-stepped process [23]. This was summarised in a series of tables and graphical representations. Findings relating to effectiveness were summarised based on the intervention content. Implementation outcomes were presented based on common themes. |
|  | 13f | Describe any sensitivity analyses conducted to assess robustness of the synthesized results. | N/A |  |
| Reporting bias assessment | 14 | Describe any methods used to assess risk of bias due to missing results in a synthesis (arising from reporting biases). | N/A |  |
| Certainty assessment | 15 | Describe any methods used to assess certainty (or confidence) in the body of evidence for an outcome. | N/A |  |
| **RESULTS** | | |  |  |
| Study selection | 16a | Describe the results of the search and selection process, from the number of records identified in the search to the number of studies included in the review, ideally using a flow diagram. | P. 7 and Figure 1 | A total of 42 papers that represented 39 interventions across 18 LMICs were included (Fig 1). A total of 38,267 studies were identified by our search strategy; 15,152 were excluded as they were duplicates (n=15,128) or were journals instead of individual papers (n=24). The titles and abstracts of the remaining 23,215, were screened, 730 were eligible for full-text screening. Seventeen additional papers were identified from reference searching and ten were recommended by our independent experts. Forty-two papers were eligible and included in our review.  Attached as Figure 1: Flow chart for selection of studies in the review |
|  | 16b | Cite studies that might appear to meet the inclusion criteria, but which were excluded, and explain why they were excluded. | N/A |  |
| Study characteristics | 17 | Cite each included study and present its characteristics. | P. 8 and Table 1 | A summary of the 42 eligible studies (sample size range from 8-2,952 participants) and their characteristics is shown in Table 1. Studies were conducted between 1998 and 2022. Six were RCTs, seven quasi-experimental, five cohort, 20 cross-sectional and four case series. The studies were conducted in 19 countries in Asia (n=31), Africa (n=14), South America (n=3), and North America (n=2), with some being multi-country. Half of the interventions targeted people experiencing mental health problems (n=18), of which four focused on pregnant women. The other half focused on general population and people experiencing mental health problems, of which six focused on children, and adolescents.  Attached as Table 1: Characteristics of included studies and interventions |
| Risk of bias in studies | 18 | Present assessments of risk of bias for each included study. | P. 20 and S6 Table | The studies were generally of mixed quality except for RCTs and quasi-experimental studies, which proportionally received least negative appraisals on the items. The mixed score was predominantly due to a high level of unclarity or lack of details, and often, a ‘not applicable’ score, which has been discussed in the limitations. For cross sectional and cohort studies, reporting on confounding factors and strategies to deal with those was rarely found. For cross sectional, the information regarding the objectivity of the outcome measure and how the measurement was conducted was in most studies unclear. For case series studies, the research sites (e.g., prevalence or details about the population) were mostly not described sufficiently and there was a lack of information on statistical analysis. See S5 Tables for assessment of all studies.  Attached as S6 Table: Risk of Bias results |
| Results of individual studies | 19 | For all outcomes, present, for each study: (a) summary statistics for each group (where appropriate) and (b) an effect estimate and its precision (e.g. confidence/credible interval), ideally using structured tables or plots. | Table 1 | Attached as Table 1: Characteristics of included studies and interventions |
| Results of syntheses | 20a | For each synthesis, briefly summarise the characteristics and risk of bias among contributing studies. | P. 8-11 | The interventionsIntervention categories Interventions were grouped into three categories following the broad steps in the help-seeking process: (1) raising mental health awareness (74% of the interventions included a component targeting this goal), (2) identification of individuals experiencing mental health problems (76%), and (3) promoting help-seeking among people identified as in need of care (64%). The majority of interventions (80%) included components in a combination of the aforementioned categories (see Table 2).  *Table 2 here Intervention content Each intervention included a varying number of components (i.e., activities, elements or techniques described in the paper) (see Table 3). The mean number of components per intervention was four (ranging from 1 to 10 components).    For raising mental health awareness, 17 different intervention components were found. The primary aim was to increase knowledge about mental health problems and available services. Most components targeted the general population and included distribution of printed materials in the community such as posters and leaflets (32,33) and social interactions and, house-to-house campaigns (34,35).  Six intervention components were used for identification of individuals experiencing mental health problems. Most frequently reported components included community- or facility-level systematic screening, case detection and online self-screening. Case detection activities were different from systematic screening as they were based on proactive identification by non-specialists (36,37). Furthermore, five studies used a tool developed to support proactive detection (i.e., the Community Informant Detection Tool [CIDT] (38)).  Nine help-seeking promotion components were identified and these were always coupled with an awareness raising and/or an identification activity. For example, sending text messages or calling people as a reminder where to find mental health care followed by messages to motivate patients to seek help (39,40) or to provide a paper-based referral card (41) about treatment options, mostly after a screening activity. Psycho-education was another common activity wherein a trained non-specialist worker, a community mental health worker (CMHW) for example, visited the house and helped the patient and their family better understand mental health problems, how to support the patient, and address any doubts concerning the treatment (33,42). Some interventions set up support groups with peers or other users of services (such as the ‘consumer advocacy group’ in South Africa) as an activity to enhance collective efficacy and help-seeking behaviour (43). In some interventions with a help-seeking promotion component the identification component was either not described or included as a study procedure. Furthermore, one-third of the interventions that included a help-seeking promotion component also included a follow up component such as home visits or phone calls to ensure adherence, overcome barriers and provide continuing psychosocial support to people with mental health problems (38,44,53).  One intervention was a policy reform introducing public health administration for immigrant workers to increase access to mental health care but did not describe specific components in the above-mentioned categories (45).  Two-thirds of the help-seeking interventions (69%; n=29) combined the above-mentioned demand-generating activities with a service provision intervention to improve access (see S4 Table). This included integrating mental health care into PHC or in schools, setting up community outreach clinics or 24-hour mental health helplines and offering online treatment using video or text messages with a mental health worker or chatbot. In addition, social support needs were also addressed by establishing links with other existing community resources, income generation opportunities and addressing the socio-economic factors that affect help-seeking (34).  *Table 3 here Implementation of the interventionsWhere: type of setting Over 70% of the interventions (n=30) were implemented in-person; mainly in the community (n=21), PHCs or community centres (n=4), or in schools (n=5). Others were delivered online (n=5), through a chatbot (46,47), a website (48), or via direct messages using social media (49,50), via media advertisement (n=3) (44,51,52) or automated text messages (n=1) (40). One provided both in-person information and reminders via the phone (53). Who: delivery agent For the interventions that were delivered in person, the most common delivery agents were non-specialist community health workers (CHWs) (n=15) which consisted of members from formal health or affiliated systems, as employees or volunteers. This included health extension workers and village health workers (VHWs). Other delivery agents included non-state providers (NSPs) outside the health sector (e.g., teachers) (n=4), NSPs in the health sector (e.g., PHCPs) (n=2), mental health workers (e.g., psychiatric social worker) (n=2), peer workers (n=1) or a combination of these (n=6). Peer workers included women, mothers, youth group members, or traditional healers, often selected based on criteria such as being motivated, respected and trusted in the community with basic literacy skills. In addition, five interventions were automated, either by sending alert messages with screening results (40,49) or using machine learning to drive chats (46,47,50). What: help-seeking source The majority of interventions promoted help seeking through formal care, at a PHC centre (n=23), community outreach clinics (n=3), hospital (n=1), school (n=1) or a combination of these (n=2) (37,54). The rest promoted remote mental health care via text messaging (n=2) (46,50) or a helpline (n=6) (44,51–53,55,56). Two of these helplines were set up in response to mental health needs due to the COVID-19 pandemic (51,56), for example through providing access to a program phone to connect with counsellors (56). Two helplines combined counselling over the phone, with home visits or online video consultations based on individual needs (44,51). Two interventions promoted self-help through participation in support groups (43) or through learning certain skills to help cope with daily stress (48). Collaboration with traditional healers in promoting help-seeking was mentioned in two studies (41,57). |
|  | 20b | Present results of all statistical syntheses conducted. If meta-analysis was done, present for each the summary estimate and its precision (e.g. confidence/credible interval) and measures of statistical heterogeneity. If comparing groups, describe the direction of the effect. | P. 12-17 | Effectiveness of interventions to increase help-seeking The results are organised by intervention category as presented in Table 2. The first two categories exclusively apply awareness raising or identification components, the remaining four use multiple categories of intervention components. The results are further organised based on their effects: (1) positive, significant; (2) positive, not significant; (3) positive trend favouring the intervention but lacking data to evaluate effect sizes; and (4) no effect. Four help-seeking related outcomes were reported: mental health service-utilisation (n=26 of 42; 62%; e.g., health centre visits, engagement with a service, or initiation of treatment); help-seeking behaviour (n=8; 19%), contact coverage (n=6; 14%) and help-seeking efficacy (n=2; 5%). Raising awareness-only Four studies evaluated interventions that exclusively targeted awareness raising (42,58–60) – all of which were cultural adaptations of the same school-based mental health literacy curriculum (61). One trial (59) showed non-significant positive results in self-reported help-seeking behaviour pre- and post-curriculum. Two studies demonstrated a positive trend in help-seeking behaviour among students and teachers; 9% of the students reached out to teachers regarding a mental health concern (60), and 63% of the teachers personally sought help post-training (42). Both combined the program with a supply-side component to integrate mental healthcare into PHC. The fourth study only reported a total number of 122 students seeking help after teacher referral of which 75% received a mental health-related diagnosis, however no denominator was reported (58). Identification-only Four studies evaluated interventions exclusively using identification components. One quasi-experimental study that evaluated a mental health training and support programme incorporated into PHC practice reported significant increase in service utilisation (p<0.05); however, this was true for both the intervention and control (62). Two studies demonstrated a positive trend in help-seeking behaviour. A pre-pilot of an artificial intelligence (AI) driven mental health intervention for pregnant women in Kenya demonstrated that at least 66% of participants sent one message to register the application and half of those continued to engage beyond registration (46). The other was a cross-sectional study in Ethiopia and reported a contact coverage of 81% (300 accessed care of 369 probable cases) after training CHWs in identification and referral (63). Both interventions combined these demand-generating activities with a supply-side component. A case series in Nepal evaluating proactive case detection using the vignette-based tool (CIDT) combined with SMS functionalities to liaise among service providers found that only 25% (2 out of 8 referred) of the people identified sought treatment (64). Raising awareness, identification, and help-seeking promotion Sixteen studies evaluated a combination of intervention components targeting awareness raising, identification and help-seeking promotion; four showed a significant increase in service utilisation. The effectiveness of raising community awareness, proactive case detection and help-seeking encouragement using a vignette-based tool (CIDT) was compared to awareness raising only in an RCT in Nepal. The median number of patients registered was 47% greater after 6 months at facilities where proactive case detection was implemented (309 accessed care in the CIDT training arm vs 182 in standard training arm; p=0.04, r=0.42) (38). A quasi-experimental study evaluated the SMART mental health project in India, which combined a help-seeking intervention comprising of an anti-stigma campaign, technology enabled screening and follow up messages for patients and a supply-side intervention of integrating mental health care into PHC. Self-reported service utilisation increased from 3.3% at baseline to 81.2% at follow up after 12 months of implementing the intervention (OR=133.3, 95% CI 89.0 to 199.7; p<0.001) (65). Two cross-sectional studies reported a significant increase in contact coverage. The first evaluated a community sensitisation program which included activities like wall paintings, detection by CHWs and provision of mental health first aid to encourage help-seeking (54). Contact coverage increased six times over 18 months (from 4.3% at baseline to 27.2% at follow up; p<0.001). The second evaluated community sensitisation, proactive case detection using the CIDT and help-seeking encouragement (66). Contact coverage based on service utilisation data over 12 months increased from 0% at baseline to 7.5% at follow up for AUD, from 0 to 12.2% for depression, from 1.3 to 11.7% for epilepsy and from 3.2 to 50.2% for psychosis. Both interventions combined the demand-generating activities with mental health care integrated into PHC. A cross-sectional study evaluating proactive case detection using the CIDT combined with mass sensitisation, stigma reduction, and help-seeking encouragement showed a non-significant increase in contact coverage (67). Contact coverage for depression increased from 8.1% at baseline to 11.8% at follow up and from 5.1% at baseline to 10.3% for AUD. A quasi-experimental study of a national program to integrate mental health care into PHC showed that provision of mental health screenings led to more people from the general population utilising mental health services and being diagnosed. Utilisation after the intervention was significantly higher in the case (19.5) than control districts (13.1) (p<0.000). However, among patients with mental disorders there was no significant increase in utilisation (68).  Eight studies showed a positive trend towards the intervention, however, did not provide data to evaluate effect sizes. Two evaluated the SMART mental health program and both showed an increase in service use (35,69). Four studies evaluated helplines that provided remote mental healthcare or referral to other services (44,51,55,70) and three reported an increase in service utilisation (51,55,70). One showed that over 70% contacted the help-line, however, only 16% sought help after being advised to do so (44). One RCT evaluated a brief educational and telephone contact program that aimed to encourage individuals who had attempted suicide to seek help. In the intervention group 27% (n=35) used the service, compared to 2% (n=2) in the TAU group (53). A chatbot introduced during COVID-19 was able to connect nearly all users who accepted to be contacted to care (42,932/42,933; 99.9%) (47) Raising awareness and identification Six studies evaluated the combination of awareness raising and identification (32,36,48, 71,72,73), and three showed a significant increase in help-seeking (71,72,73). One stepped-wedge cluster RCT evaluated the added impact of a classroom sensitization session compared to whole-school sensitization activities on demand for a school counselling service for adolescents (73). The proportion of students referred in the former was significantly higher (21.7% vs 1.5%, OR=111.36, 95% CI 35.56 to 348.77, p<0.001) (73). The proportion of (self-) referred students was also higher in the classroom sensitization arm (73). Two evaluated a mental health program in Nigeria which included awareness raising using local media and posters along with training of VHWs in identification and referral of people experiencing mental health problems to services integrated into PHC. A quasi-experimental study (72) showed a five times higher incidence rate of new patients, sustained for over a year after the intervention. The cross-sectional study (71) showed that the increase occurred with a strong temporal relationship to the training of VHWs and increases were mainly sustained for the following month, with a tail off from the very high levels immediately following the training. Meanwhile, another cross-sectional study in Uganda with similar intervention components, but specifically targeting depression and alcohol use disorder (AUD) found a non-significant increase in contact coverage (ranging from 1.3 to 4.1%) (36). A cross-sectional study in India studied the impact of a mental health awareness campaign targeting the general public. It comprised of disseminating educational materials, conducting public meetings, street plays and quizzes, and organising screening camps. Nearly 66% received a preliminary diagnosis, indicating the success of campaign messages in improving recognition of mental health problems (32). Finally, a cross-sectional study evaluating a website launched for secondary school students with information on mental health and psychological wellbeing showed that nearly all of the students (98.6%) visited the website (48) and 10- 20% also used it for other reasons like seeking help and sharing information. Raising awareness and help-seeking promotion Five studies evaluated a combination of intervention components targeting awareness raising and help-seeking promotion. Only one showed a significant increase in number of patients initiating treatment. This cross-sectional study evaluated the use of educational materials and a community-led and peer-led support system (e.g., a closed Facebook page) combined with providing facility-based one-on-one counselling, specifically for the transgender population in India. Access to mental health support increased from 43.7% at baseline to 77.2% at end line (p<0.001) (34). Another cross-sectional study evaluated a focal point programme providing linkages to mental healthcare via a project phone during COVID-19, and 332 (48.9%) of the 679 visits resulted in a phone call to a counsellor. A cross-sectional study about a mental health support group in South Africa found that 64% of the participants rated the group to be helpful in encouraging help-seeking (43). A feasibility trial in Pakistan evaluated psychoeducation by a lady health worker (LHW) at home and resulted in 71% of women or their family members contacting a LHW for assistance within 2 months of intervention completion, compared to 46% in the control arm (p=0.036) (33). Finally, a cross-country evaluation of case detection by trained facility workers in India, Nepal, Ethiopia, South Africa and Uganda found low to no change in treatment initiation for those who screened positive for depression or AUD (74). Identification and help-seeking promotion Six studies evaluated interventions that combined identification with help-seeking promotion, out of which two RCTs reported a significant increase in help-seeking behaviour. One trial compared the effectiveness of enhanced care by CHWs with regular referrals. Enhanced care included regular home visits and psycho-education with the target population and their family members. Participants in the enhanced care arm were two times more likely to visit the clinic as compared to treatment as usual and a significantly higher number of visits were found in the enhanced care arm (p<0.001) (75). A cohort study in Nepal, using a similar community-based approach, evaluated proactive case detection using the CIDT in combination with help-seeking encouragement by key community members (41). About 67% of the people who were referred sought help, however no data was presented to evaluate effect sizes. The second RCT evaluated online self-screening and automated help-seeking promotion messages. They assessed the effect of intensity of prompts in the messages on help-seeking behavior (49). This study supported the use of a stronger, more direct prompt message post screening rather than a ‘lighter’ one. Across all six countries, 10.2% reported seeking treatment after the light prompt at follow up and 16.6% following the strong prompt (p<0.002). A similar approach of sending help-seeking promotion messages was used in three other studies, out of which two demonstrated a promising trend in help-seeking behaviour. One combined a mental health screening app used by PHCPs with automated motivational text messages; over 70% of the patients receiving the message sought help (39). Another cohort study evaluated identification of suicide risk using a machine learning model combined with automated messages with help-seeking options. About 44.4% of the people responded (50). A cohort study evaluating the effects of help-seeking promotion text messages for perinatal depression in China, showed that only 1.2% reached out for further information and only one-third of them attended an appointment after receiving information (40).  One intervention was a policy reform for immigrant workers to increase access to mental health care (45). The intervention components fell outside of the above created categories. But, a path model analysis found that introducing public health administration and the community policy reform had the most direct impact on mental health service utilisation (p<0.01). |
|  | 20c | Present results of all investigations of possible causes of heterogeneity among study results. | N/A |  |
|  | 20d | Present results of all sensitivity analyses conducted to assess the robustness of the synthesized results. | N/A |  |
| Reporting biases | 21 | Present assessments of risk of bias due to missing results (arising from reporting biases) for each synthesis assessed. | N/A |  |
| Certainty of evidence | 22 | Present assessments of certainty (or confidence) in the body of evidence for each outcome assessed. | N/A |  |
| **DISCUSSION** | | |  |  |
| Discussion | 23a | Provide a general interpretation of the results in the context of other evidence. | P. 21-24 | Trends in intervention content and effectiveness Ten studies (24%) showed a statistically significant increase in help-seeking behaviour (34,38,49,54,65,66,71–73,75). This is encouraging given the few trials found in LMICs in previous systematic reviews (19,20,77). Help-seeking intervention research often use two main (separate) approaches; universal interventions aimed at the general population targeting attitudes and intentions, and indicated interventions targeted at those who experience mental health problems (19,77). Previous reviews found that interventions that improved attitudes and intentions, did not necessarily lead to improved help-seeking behaviour (19); in line with this, none of the interventions which only focused on information sharing activities were found to improve help-seeking behaviour (77). We found a similar pattern for interventions that exclusively used awareness raising components for example. Although the studies showed a positive trend in help-seeking behaviour, the results were not significant or lacked data to evaluate effectiveness. Hence, to have a population level effect on help-seeking, a combined approach was recommended in the prior review (19). The current review shows a similar trend; all ten studies that yielded a statistically significant effect on help-seeking behaviour at post intervention targeted multiple categories.  Out of the ten studies with positive significant findings, all but one (i.e., an online intervention) (49), involved non-specialists like CHWs and peer workers as the primary delivery agents. They were often selected as trusted and engaged individuals, with regular contact with community members or students. This strategy may be especially beneficial in areas where mental health literacy is low, and where formal health services are not necessarily seen as a place to seek treatment for mental health problems. Furthermore, community members use shared cultural idioms which could promote more effective communication, facilitate trust and therefore lower barriers to seeking help and ultimately, reduce stigma (78,79). These in-person interventions all linked people to mental healthcare based in the community, and one in schools (73). This integration of mental healthcare into community contexts like PHC follows recommended models to tackle supply-side barriers such as accessibility and costs of services (3,80).  Two RCTs demonstrated that a low-intensity intervention using the task-sharing approach resulted in significantly more number of visits to mental health care. Jordans and colleagues (38) used a vignette-based tool to support proactive community level detection by key community members; the median number of patients registered was 47% greater after 6 months. Pradeep and colleagues (75) found that CMHWs could effectively monitor and psycho-educate patients to improve help-seeking. Participants were two times more likely to visit the clinic as compared to treatment as usual. From a public health perspective, these results are promising especially given the low-intensity of the interventions and the use of the task-sharing approach. These results are consistent with findings that showed that psychological treatments using a limited number elements were effectively delivered by non-specialist providers with moderate to strong effects sizes (81).  Two quasi-experimental studies with statistically significant results showed an increase in help-seeking behaviour of 5 (72) to 23 times higher (65) over the course of one year. The intervention with the greatest impact (i.e., the SMART mental health project) also appears to be of highest intensity. The intervention focused on strengthening collaboration between implementation agents and service providers and tailored pre-recorded messages were sent to screen-positive individuals, ASHAs and doctors (65). However, this study also reported that only 33% of the cases identified by CMHWs were clinically diagnosed with a mental health problem. Although this did not pose an issue in this study since there was sufficient availability of services and could be explained by natural remission, in other settings this could pose a potential risk of overburdening the health system. Following the hypothesised importance of the perceived need for mental health care as a mediator for actual help-seeking (82), further optimising the accuracy of identification may boost effectiveness of help-seeking interventions [71]. Another important caveat is that only Eaton et al. (72) assessed long-term impact and showed that the effect gradually tailed off after one year. Similar challenges in sustaining the results (e.g., drop in detection or app use) was highlighted by other studies (36,46). This may especially pose a challenge for interventions that apply task-sharing approaches or work with community members as it often results in increased work pressure and challenges in sustaining motivation (83). Recommended ways to improve motivation and continued engagement are substantial investments in all layers of staff, regular supervision meetings and, structured refresher trainings (37,71,72). However, the reliance of most help-seeking interventions on this group warrants further investigation on sustainability of interventions from their perspective.  Another element that appears to be promising is the use of new technology to send digital prompts (e.g., text or online messages). Two tech-enabled interventions showed a significant increase in help-seeking behaviour. One is described above, and included sending pre-recorded messages to screen-positive individuals, ASHAs and doctors. This active follow up resulted in adherence of nearly 100% (65). The other showed that stronger help-seeking promotion messages generally resulted in greater likelihood of seeking care (49). This is especially promising as increasingly, more people have access to a digital device and search for health information online (6). Previous systematic reviews, mainly in HICs, found mixed results regarding the use of new technology. Gulliver et al. (77) for example, found two interventions conducted online (i.e., via email and a website plus telephone contact) both of which successfully increased formal help-seeking while Xu et al. (20) found no effectiveness for internet-based interventions and only short-term effects for telephone-based interventions. Target population Given the influences of social networks on help-seeking behaviour, the interventions in our review not only focused on individuals with mental health problems, but also on important gatekeepers, such as family members or teachers, who can recognise symptoms and ‘open the gate’ to support [20].  Six studies (14%) focused on children and adolescents out of which only one reported a significant increase in help-seeking (73). This classroom sensitisation program in India was delivered by a lay counsellor and used short animated videos with moderated group discussions. During the time periods when the classroom sessions were delivered, the referral proportion rose significantly. According to this and a previously conducted systematic review (20) - that found no effect for interventions targeting children - this is the only help-seeking promotion intervention for adolescent mental healthcare yielding a statistically significant effect on help-seeking. Two-thirds of the students that sought help in this trial did not meet the clinical thresholds for symptom severity, functional impact and chronicity, and further research may therefore be needed to avoid over-detection. Furthermore, school-based programs like these will need to be coupled with school-counselling services and suitable interventions for adolescents who do not meet clinical thresholds.  Studies also showed different impact of the same intervention based on the mental health problem that was targeted or the gender of the target population. For example, contact coverage increased by 8% for AUD but 50% for psychosis (66), and another study showed that adult men were not adequately targeted by the intervention (36). Different strategies may therefore be beneficial based on the target group. |
|  | 23b | Discuss any limitations of the evidence included in the review. | P. 25 | The review only included papers published in English. Due to the heterogeneity in outcome measures and study designs, only limited conclusions on the effectiveness of strategies could be drawn. |
|  | 23c | Discuss any limitations of the review processes used. | P. 26 | In addition, the intervention activities were extracted and included only if they were explicitly reported in the publication. Lastly, inter-rater reliability for the abstract screening phase was not assessed. |
|  | 23d | Discuss implications of the results for practice, policy, and future research. | P. 25 | Although this review shows a promising trend for the effectiveness of strategies increasing help-seeking behaviour, there has been a limited number of robust studies undertaken in LMICs. This review has highlighted that especially interventions which used a combination of intervention components resulted a statistically significant effect on help-seeking behaviour. It is therefore recommended that future interventions include various activities targeting awareness-raising among the general population, identification of individuals experiencing a mental health problem and specific help-seeking promotion. Furthermore, research on long-term effects of the intervention as well as sustainability (in terms of maintaining motivation and providing compensation to delivery agents) needs to be further explored. Moreover, it should be acknowledged that even if effective help-seeking interventions exist and utilisation increases, this does not guarantee a positive effect on mental health outcomes. A combination of demand (e.g., user-level factors) and supply-side factors (e.g., facility-, provider- level factors) need to be studied to understand the success in clinical effects. In other words, we need to develop a better understanding on how the help-seeking process works, and which combination of demand- and supply-side interventions are most effective in LMICs. Some studies pointed out to the importance of culture in the help-seeking process. For example, one study explained low uptake by placing the emphasis on joint decision making for a family member experiencing mental health problems (40). Areas for further exploration also include involvement of family members and people with lived experiences, which is a central active ingredient in interventions to reduce stigma, enhance the demand for services (86) and on interventions focussing on children and adolescents. |
| **OTHER INFORMATION** | | |  |  |
| Registration and protocol | 24a | Provide registration information for the review, including register name and registration number, or state that the review was not registered. | P. 4 | This systematic review was conducted in accordance with PRISMA (Preferred Reporting Items for Systematic Reviews and Meta-Analyses) guidelines (see S2 Table) [24]. The protocol was registered a priori (Registration number: CRD42021255635; S3). |
|  | 24b | Indicate where the review protocol can be accessed, or state that a protocol was not prepared. | P. 4 and S3 | This systematic review was conducted in accordance with PRISMA (Preferred Reporting Items for Systematic Reviews and Meta-Analyses) guidelines (see S2 Table) [24]. The protocol was registered a priori (Registration number: CRD42021255635; S3). |
|  | 24c | Describe and explain any amendments to information provided at registration or in the protocol. | N/A |  |
| Support | 25 | Describe sources of financial or non-financial support for the review, and the role of the funders or sponsors in the review. | P. 26 | This review was conducted as a part of a research study called IMPlementation of evidence-based facility and community interventions to reduce the treatment gap for depRESSion (IMPRESS). The grant was awarded by National Institute of Mental Health to Sangath [Grant number: RO1MH115504]. War Child Holland was supported by a foundation that wishes to remain anonymous. The foundation had no role in the study design and implementation. |
| Competing interests | 26 | Declare any competing interests of review authors. | N/A |  |
| Availability of data, code and other materials | 27 | Report which of the following are publicly available and where they can be found: template data collection forms; data extracted from included studies; data used for all analyses; analytic code; any other materials used in the review. | N/A |  |

**Table 1** PRISMA checklist (2020)
