# Supplementary material for: Interventions to increase help-seeking for mental health care in low- and middle-income countries: A systematic review
Source: PLOS Glob Public Health. 2023 Sep 13;3(9):e0002302. doi: 10.1371/journal.pgph.0002302 (PMC10499262; doi:10.1371/journal.pgph.0002302)
Supplement: S2 Table — (DOCX) [file pgph.0002302.s002.docx]

**S2 Table. Supply-side components**

|  | Integration of mental health into PHC | Integration of mental health into schools | Linking people to community resources | Outreach community-based care | Setting up helplines for treatment | Remote treatment | Remote automated treatment | Policy reform | Establishing two-way communication | Including people with lived experiences | Collaborating with traditional healers | Engaging key community members | Coordinating with media | Including PHC staff during trainings | Building literacy among teachers | For awareness/ support | To detect and refer people to services | Capacity strengthening for health care staff |
| --- | --- | --- | --- | --- | --- | --- | --- | --- | --- | --- | --- | --- | --- | --- | --- | --- | --- | --- |
| **Author(s) (year)** | **Service delivery** | | | | | | | **System** | | **Involve stakeholders** | | | | | **Training** | | | |
| Bhardwaj et al. (2020) |  |  |  |  |  |  |  |  | • |  |  | • |  |  |  |  | • | • |
| Byaruhanga et al. (2008) |  |  |  | • |  |  |  |  | • | • | • | • |  |  |  | • |  | • |
| Chavan et al. (2012) |  |  |  |  | • |  |  |  |  |  |  |  | • |  |  |  |  |  |
| Diez-Canseco et al. (2018) | • |  |  |  |  |  |  |  |  |  |  |  |  |  |  |  |  | • |
| Eaton et al. (2008) |  |  |  |  |  |  |  |  |  |  |  | • |  | • |  | • | • |  |
| Eaton et al. (2017) |  |  |  |  |  |  |  |  |  |  |  | • |  | • |  |  | • |  |
| Gaiha et al. (2021) |  |  |  |  |  |  |  |  |  |  |  | • |  |  |  |  |  |  |
| Gong et al. (2020) |  |  |  |  |  |  |  |  |  |  |  |  |  |  |  |  |  |  |
| Green et al. (2020) |  |  |  |  |  |  | • |  |  |  |  |  |  |  |  |  |  |  |
| Hailemariam et al. (2019) | • |  |  |  |  |  |  |  |  |  |  | • |  |  |  |  | • | • |
| Hajebi et al. (2021)^1^ | • |  |  |  |  |  |  |  |  |  |  | • |  |  |  |  |  | • |
| He et al. (2020) |  |  | • |  | • | • |  |  |  |  |  |  |  |  |  |  |  | • |
| James et al. (2002) | • |  |  |  |  |  |  |  |  |  |  |  |  |  |  |  |  | • |
| Jordans et al. (2017) |  |  |  |  |  |  |  |  |  |  |  | • |  |  |  |  | • |  |
| Jordans et al. (2019)^2^ | • |  | • |  |  |  |  |  |  |  |  | • |  |  |  |  | • | • |
| Jordans et al. (2020) |  |  |  |  |  |  |  |  |  |  |  | • |  |  |  |  | • |  |
| Kaewanuchit et al. (2019) |  |  |  |  |  |  |  | • |  |  |  |  |  |  |  |  |  |  |
| Khan et al. (2017) |  |  |  |  |  |  |  |  |  |  |  | • |  |  |  | • |  |  |
| Kutcher et al. (2016) | • |  |  |  |  |  |  |  |  |  |  |  |  |  | • |  |  | • |
| Kutcher et al. (2017^a^) | • |  |  |  |  |  |  |  |  |  |  |  |  |  | • |  |  | • |
| Kutcher et al. (2017^b^) |  |  |  |  |  |  |  |  |  |  |  |  |  |  | • |  |  | • |
| Lee et al. (2022) |  |  |  |  |  | • |  |  |  |  |  | • |  |  |  |  | • |  |
| Leykin et al. (2013) |  |  |  |  |  |  |  |  |  |  |  |  |  |  |  |  |  |  |
| Liu et al. (2019) |  |  |  |  |  | • |  |  |  |  |  |  |  |  |  |  |  |  |
| Luitel et al. (2019)^2^ | • |  | • |  |  |  |  |  |  |  |  | • |  |  |  |  | • | • |
| Malakouti et al. (2022) |  |  |  |  | • |  |  |  |  |  |  |  |  |  |  |  |  |  |
| Maulik et al. (2017) | • |  |  | • |  |  |  |  | • | • |  | • |  |  |  |  | • | • |
| Maulik et al. (2020) | • |  |  | • |  |  |  |  | • | • |  | • |  |  |  |  | • | • |
| Nakku et al. (2019)^3^ | • |  |  |  |  |  |  |  |  |  |  | • |  |  |  | • | • | • |
| Nguyen et al. (2021) |  |  |  |  |  |  |  |  |  |  |  |  |  |  |  |  |  |  |
| Parikh et al. (2021) |  | • |  |  |  |  |  |  |  |  |  |  |  |  |  |  |  |  |
| Pradeep et al. (2014) | • |  |  |  |  |  |  |  |  |  |  | • |  |  |  | • |  | • |
| Ragesh et al. (2020) |  |  |  |  | • |  |  |  |  |  |  |  |  |  |  |  |  |  |
| Rathod et al.(2018)^4^ | • |  |  |  |  |  |  |  |  |  |  | • |  |  |  | • | • | • |
| Ravindran et al. (2018) |  |  |  |  |  |  |  |  |  |  |  |  |  |  | • |  |  |  |
| Shaikh et al. (2016) |  |  | • | • |  |  |  |  |  | • |  |  |  |  |  |  |  |  |
| Shidhaye et al. (2017) | • |  |  |  |  |  |  |  |  |  |  | • |  |  |  |  | • |  |
| Shidhaye et al. (2019)^5^ | • |  | • | • |  |  |  |  |  |  |  | • |  |  |  |  |  | • |
| Shrivastava et al. (2012) |  |  |  | • | • |  |  |  |  |  |  |  |  |  |  |  |  |  |
| Stein et al. (2001) |  |  |  |  |  |  |  |  |  |  |  |  |  |  |  |  |  |  |
| Tewari et al. (2017) | • |  |  | • |  |  |  |  | • | • |  | • |  |  |  |  | • | • |
| Tzelios et al. (2022) |  |  |  |  |  |  |  |  | • |  |  |  |  |  |  |  |  |  |
| Total | 16 | 1 | 5 | 7 | 5 | 3 | 1 | 1 | 6 | 5 | 1 | 21 | 1 | 2 | 4 | 6 | 15 | 19 |

**Table 2**. Supply-side components

Note: Papers that were referenced and used in this review for the intervention description are: ^1^[1]; ^2^[2]; ^3^[3]; ^4^[4]; ^5^[5].
Kutcher et al. (2017a) [6]; Kutcher et al. (2017b) [7]

**References**

1. Damari B, Sharifi V, Asgardoon MH, Hajebi A. Iran’s Comprehensive Mental and Social Health Services (SERAJ Program): A Pilot Protocol. 2021.

2. Jordans MJD, Luitel NP, Pokhrel P, Patel V. Development and pilot testing of a mental healthcare plan in Nepal. British Journal of Psychiatry. 2016;208: s21–s28. doi:10.1192/bjp.bp.114.153718

3. Kigozi FN, Kizza D, Nakku J, Ssebunnya J, Ndyanabangi S, Nakiganda B, et al. Development of a district mental healthcare plan in Uganda. British Journal of Psychiatry. 2016;208: s40–s46. doi:10.1192/bjp.bp.114.153742

4. Petersen I, Fairall L, Bhana A, Kathree T, Selohilwe O, Brooke-Sumner C, et al. Integrating mental health into chronic care in South Africa: The development of a district mental healthcare plan. British Journal of Psychiatry. 2016;208: s29–s39. doi:10.1192/bjp.bp.114.153726

5. Shidhaye R, Shrivastava S, Murhar V, Samudre S, Ahuja S, Ramaswamy R, et al. Development and piloting of a plan for integrating mental health in primary care in Sehore district, Madhya Pradesh, India. British Journal of Psychiatry. 2016;208: s13–s20. doi:10.1192/bjp.bp.114.153700

6. Kutcher S, Wei Y, Gilberds H, Brown A, Ubuguyu O, Njau T, et al. The African Guide: One Year Impact and Outcomes from the Implementation of a School Mental Health Literacy Curriculum Resource in Tanzania. J Educ Train Stud. 2017;5: 64. doi:10.11114/jets.v5i4.2049

7. Kutcher S, Udedi M, Gilberds H, Brown A, Chapota R, Perkins K. Clinic outcomes of the pathway to care model: A cross-sectional survey of adolescent depression in Malawi. Malawi Medical Journal. 2017;29: 97–102. doi:10.4314/mmj.v29i2.4
