# Supplementary material for: Interventions to increase help-seeking for mental health care in low- and middle-income countries: A systematic review
Source: PLOS Glob Public Health. 2023 Sep 13;3(9):e0002302. doi: 10.1371/journal.pgph.0002302 (PMC10499262; doi:10.1371/journal.pgph.0002302)
Supplement: S3 Table — (DOCX) [file pgph.0002302.s003.docx]

**S3 Table. Risk of Bias results**

| **Author** | **Q1** | **Q2** | **Q3** | **Q4** | **Q5** | **Q6** | **Q7** | **Q8** | **Q9** | **Q10** | **Q11** | **Q12** | **Q13** |
| --- | --- | --- | --- | --- | --- | --- | --- | --- | --- | --- | --- | --- | --- |
| Jordans et al. 2020 | Y | Y | U | N/A | N/A | N/A | Y | N/A | Y | Y | U | U | U |
| Khan et al. 2017 | Y | N | Y | U | N | Y | Y | Y | Y | Y | U | Y | U |
| Leykin et al. 2013 | U | U | U | U | U | U | U | U | Y | Y | U | Y | U |
| Malakouti et al. 2022 | Y | Y | U | Y | Y | U | Y | Y | Y | Y | Y | Y | U |
| Parikh et al. 2021 | Y | Y | U | N/A | N/A | N/A | Y | Y | U | Y | U | Y | U |
| Pradeep et al. 2014 | Y | U | Y | N/A | N/A | Y | Y | Y | Y | Y | U | Y | Y |

**Table 3** Critical appraisal results for included RCTs using the JBI Critical Appraisal Checklist

| **Author** | **Q1** | **Q2** | **Q3** | **Q4** | **Q5** | **Q6** | **Q7** | **Q8** | **Q9** |
| --- | --- | --- | --- | --- | --- | --- | --- | --- | --- |
| Eaton et al. 2017 | Y | U | U | N | U | U | U | U | U |
| Hajebi et al. 2021 | Y | U | U | Y | Y | U | Y | Y | U |
| James et al. 2002 | Y | U | Y | Y | Y | U | Y | Y | Y |
| Kutcher et al. 2016 | Y | Y | U | N | Y | U | N/A | U | U |
| Maulik et al. 2017 | Y | Y | N/A | N | Y | Y | U | U | Y |
| Maulik et al. 2020 | Y | Y | U | N | Y | U | N/A | U | Y |
| Ravindran et al. 2018 | Y | U | Y | Y | Y | U | Y | U | U |

**Table 4** Critical appraisal results for included Quasi-Experimental Studies using the JBI Critical Appraisal Checklist

| **Author** | **Q1** | **Q2** | **Q3** | **Q4** | **Q5** | **Q6** | **Q7** | **Q8** |
| --- | --- | --- | --- | --- | --- | --- | --- | --- |
| Byaruhanga et al. 2008 | N | U | U | N | N | N | U | U |
| Chavan et al. 2012 | N/A | Y | U | Y | N | N | U | Y |
| Gaiha et al. 2021 | N/A | Y | Y | U | N | N | U | U |
| Hailemariam et al. 2019 | U | Y | U | Y | Y | Y | Y | Y |
| Jordans et al. 2019 | Y | Y | Y | Y | U | U | U | Y |
| Kaewanuchit et al. 2019 | Y | Y | U | Y | U | N | Y | Y |
| Kutcher et al. 2017 | U | U | U | Y | N/A | N/A | U | U |
| Lee et al. 2022 | N/A | Y | Y | U | N | N/A | Y | U |
| Luitel et al. 2019 | Y | Y | Y | Y | U | N | U | Y |
| Nakku et al. 2019 | U | Y | U | Y | Y | Y | U | Y |
| Nguyen et al. 2021 | Y | Y | Y | U | N | N/A | U | Y |
| Ragesh et al. 2020 | U | U | Y | U | N | N | U | Y |
| Rathod et al. 2018 | Y | Y | Y | Y | N | N | U | Y |
| Shaikh et al. 2016 | Y | Y | U | N/A | N | N | U | Y |
| Shidhaye et al. 2017 | Y | Y | Y | Y | U | Y | U | Y |
| Shidhaye et al. 2019 | Y | Y | Y | Y | U | U | U | Y |
| Shrivastava et al. 2012 | U | N | U | U | N/A | N/A | U | Y |
| Stein et al. 2001 | N | U | U | U | U | N | U | U |
| Tzelios et al. 2022 | N | U | U | Y | N | N/A | U | Y |

**Table 5** Critical appraisal results for included Analytical Cross Sectional Studies using the JBI Critical Appraisal Checklist

| **Author** | **Q1** | **Q2** | **Q3** | **Q4** | **Q5** | **Q6** | **Q7** | **Q8** | **Q9** | **Q10** | **Q11** |
| --- | --- | --- | --- | --- | --- | --- | --- | --- | --- | --- | --- |
| Eaton et al. 2018 | Y | U | U | N | N | U | Y | Y | U | N/A | U |
| Gong et al. 2020 | Y | Y | Y | N | N | U | U | Y | U | Y | Y |
| Jordans et al. 2017 | Y | Y | U | U | U | U | U | Y | Y | N | U |
| Kutcher et al. 2017 | U | Y | U | N | N | U | U | Y | U | N | U |
| Liu et al. 2019 | Y | U | U | N | N | N | Y | N | N | N | Y |
| Tewari et al. 2017 | Y | Y | U | N | N | U | U | U | Y | U | U |

**Table 6** Critical appraisal results for included Cohort studies using the JBI Critical Appraisal Checklist

| **Author** | **Q1** | **Q2** | **Q3** | **Q4** | **Q5** | **Q6** | **Q7** | **Q8** | **Q9** | **Q10** |
| --- | --- | --- | --- | --- | --- | --- | --- | --- | --- | --- |
| Bhardwaj et al. 2020 | N | Y | U | N | N | U | U | U | N | U |
| Diez-Canseco et al. 2018 | Y | Y | Y | U | Y | U | Y | U | U | Y |
| Green et al. 2020 | Y | Y | Y | Y | Y | Y | Y | Y | Y | U |
| He et al. 2020 | U | U | U | U | U | N | N | U | N | U |

**Table 7** Critical appraisal results for included Case Series using the JBI Critical Appraisal Checklist
