# Supplementary material for: Interventions to increase help-seeking for mental health care in low- and middle-income countries: A systematic review
Source: PLOS Glob Public Health. 2023 Sep 13;3(9):e0002302. doi: 10.1371/journal.pgph.0002302 (PMC10499262; doi:10.1371/journal.pgph.0002302)
Supplement: S1 Text — (PDF) [file pgph.0002302.s004.pdf]

Citation

Abhijit Nadkarni, Yashi Gandhi, Myrthe van den Broek. Interventions to increase help-seeking for mental health care services in low- and- middle-income countries: a systematic review. PROSPERO 2021 CRD42021255635 Available from:
https://www.crd.york.ac.uk/prospero/display_record.php?ID=CRD42021255635

Review question

The aim of this systematic review is to synthesise evidence from low- and middle-income countries (LMICs) about interventions that aim to improve help-seeking behaviour for mental health problems.

Primary objectives

1. To synthesize effectiveness of help-seeking interventions for mental health problems in improving help-seeking behaviour, service utilization and contact coverage in LMICs.

2. To describe and categorise core components and key implementation processes of the reported help-seeking interventions.

Secondary objectives

1. To synthesize information on mediating factors related to help-seeking behaviour, service utilization and contact coverage like knowledge, attitudes and intentions to seek help.

2. To synthesize information about the perceptions and implementation of the reported help-seeking interventions.

Searches

Our information sources will include:

1. Peer-reviewed published literature

This will be searched on the following databases: MEDLINE, PsycINFO, Embase, Global Health, CENTRAL, AJOL, LILACS and SciELO

2. Reference list of the systematic reviews previously conducted on this topic area will be cross-referenced:

i. Xu, Z., Huang, F., Koesters, M., Staiger, T., Becker, T., Thornicroft, G., & Ruesch, N. (2018). Effectiveness of interventions to promote help-seeking for mental health problems: systematic review and meta-analysis. Psychological Medicine, 48(16), 2658-2667.

ii. Werlen, L., Gjukaj, D., Mohler-Kuo, M., & Puhan, M. A. (2020). Interventions to improve children's access to mental health care: a systematic review and meta-analysis. Epidemiology and psychiatric sciences, 29.

3. Experts in the field will be contacted with the final inclusion list to see if they can identify any further useful published literature

4. Backward and forward citation searching of the included studies

Only research papers published in English will be included in the review. For the purpose of the search, there is no restriction on the year in which the study has been conducted or the publication period.

Types of study to be included

Inclusion: Any study presenting primary data on the outcomes of interest:

i. Experimental studies (e.g. individual randomized trials, cluster randomized trials, pragmatic trial).

ii. Quasi-experimental studies (e.g. controlled before and after designs and interrupted time series design)

iii. Non-experimental studies (e.g. cohort studies, repeat cross-sectional studies, single group pre-post -test and post-program only, case series).

Exclusion: Reviews, meta-analyses, commentaries, opinion pieces

Condition or domain being studied

Help-seeking promotion interventions for mental health problems

Participants/population

The target population could be of any age and will include:

i. The general population

ii. Individuals with any mental health problem, as

o established mental health condition following DSM or ICD

o identified using a structured questionnaire (e.g. PHQ-9)

o diagnosed by a clinician

iii. Individuals identified in the manuscript as being at risk of developing any mental health problem

Intervention(s), exposure(s)

In this systematic review we conceptualize help-seeking as a dynamic multi stepped process that starts with the awareness of a problem that may require help, followed by the expression of symptoms and need for support, the identification of accessible help sources, and finally the willingness to seek help and disclose relevant information (Rickwood et al., 2005). Any intervention with the stated aim of promoting help-seeking for any kind of mental health care will be included. The steps in this process of seeking help can be carried out by an individual who perceives herself/himself in need, or on behalf of an individual in need (e.g. caregiver, or other family member).

Comparator(s)/control

Not applicable

Context

The eligible studies must be conducted in any setting in a low- and middle-income country, as per the World Bank categorization for 2021

Main outcome(s)

1. The primary outcomes for effectiveness include:

- Help-seeking behaviour: any measure of contact with an external source of help, including formal, informal and self-help or acts of service use.

- Service utilization: any measure of the ratio between the capacity and the number of people that received the service (Tanahashi, 1978).

- Contact coverage: any measure of the proportion of persons in need of mental health treatment who receive an intervention that is appropriate to their condition (De silva et al., 2014; Tanahashi, 1978).

These outcomes can be measured at the individual and population/service level, and should be measured as a stated aim of the help-seeking promotion intervention.

2. Describing the core components and key implementation processes (e.g.: who, where, how, etc.) of the reported help-seeking interventions.

Additional outcome(s)

1. Secondary outcomes related to mediating factors of help-seeking behaviour, service utilization and contact coverage.

2. Secondary outcomes related to implementation outcomes like feasibility, acceptability, appropriateness and other related outcomes will be reported.

Data extraction (selection and coding)

1. After conducting the search based on the above outlined search strategy, all results from different sources will be imported into Covidence.

2. Duplicate records will be removed automatically by Covidence upon import. A manual check will also be undertaken by the researchers.

3. 10 percent of the papers will be screened independently by both the reviewers and the rest will be screened by only one of the reviewers. The interrater reliability (IRR) will be calculated as the percentage of agreement between the two coders for the 10 percent. After resolving conflicts and addressing clarity issues regarding the eligibility criteria, the rest of the results will be screened by one reviewer. An agreement percentage above 80% will be regarded as acceptable. A third reviewer will resolve conflicts when no consensus is reached between the two reviewers. Justification for exclusion will be recorded in Covidence and full texts of the selected potentially relevant reports will be retrieved.

4. Studies on the same intervention but conducted in another context or focusing on different outcomes will be included. In case we come across multiple studies, on the same intervention, same context, same outcomes, but different times, the most recent study will be included.

5. For the publications that were selected after review of the title and abstract, the full text of each study will be reviewed independently by two reviewers. Justification for exclusion will be recorded. Another reviewer will resolve conflicts when no consensus is reached between the two reviewers.

The process and results will be presented in a flowchart following the Preferred Reporting Items for Systematic Reviews and Meta-Analyses (PRISMA) guidelines. Three reviewers will extract data from the published reports of included studies using a standardized, pre-piloted extraction form. Any missing information will be collected by contacting the corresponding authors via email.

Risk of bias (quality) assessment

The quality and the risk of bias of all studies selected for inclusion will be assessed by two reviewers using the Joanna Briggs Institute critical appraisal tools (JBI critical appraisal checklist) (JBI, 2020). The relevant checklists for the included study designs will be used such as the checklist for randomized controlled trials, quasi-experimental studies, cohort studies, case series, case control studies and cross -sectional studies. A third reviewer will resolve disagreements between the two reviewers. An overall quality appraisal score for each study will be calculated to provide a measure of the quality (i.e. low, medium and high). Researchers will also be contacted to request information related to the study characteristics, eligibility and risk of bias that are not clear in the published information retrieved.

Strategy for data synthesis

Considering the expected heterogeneity of the study designs, variety of outcomes and expected wide range of interventions that will be included, we will provide a narrative synthesis of the findings.

First, preliminary descriptive summaries will be provided of each individual study. Summaries will include a description of selected relevant information from the data extraction sheet, including (1) the intervention type; (2) target population; (3) age; (4) mental health condition; (5) delivery method; (5) setting of delivery of the intervention; (7) study design; (8) outcome measures; (9) help-seeking outcomes; and (10) implementation related outcomes (if included in the study).

Second, sub-groups of studies will be created based on study design. Study characteristics, quality, and findings of each study within a sub-group will be summarized. Depending on the number of studies included in each sub-group and the homogeneity in outcomes assessed within each sub-group, the similarities and differences in intervention type, target population (i.e. general population, or targeted at people experiencing a mental health condition), mental health condition and outcomes between studies will be synthesised. If possible, associations will be reported between help-seeking outcomes (i.e. attitudes, knowledge, intentions and behaviour) among studies in each sub-group.

Furthermore, studies measuring the same outcomes with different designs, or among a different population, or with a different intervention type, or among different world bank classification groups, may also be grouped together and similarities, differences and patters in for example effects vis-à-vis variability in study design or setting may be explored.

Studies reporting on implementation-related outcomes will also be grouped, and similarities and differences in delivery method, setting of delivery, mental health condition, and target population will be compared against reported implementation outcomes.

Analysis of subgroups or subsets

Descriptive analysis of the data will be undertaken to address the findings of each study outcome. The results will mainly be reported in a narrative format focusing on answering each of the objectives described above. A series of tables and graphical representations will be produced to report findings relating to help-seeking behaviour, service utilization and contact coverage related outcomes of the evaluated interventions as well as the other outcomes related to help-seeking and the implementation of the interventions. Based on the availability of data and homogeneity of designs and outcomes (see data synthesis), sub-analysis may be conducted for different age groups (e.g. children, adolescents, adults), mental health conditions (e.g. developmental disorders, CMD, SMD, etc), intervention type and world bank classification group (low-income, low-middle, upper-middle country).

Contact details for further information

Abhijit Nadkarni
abhijit.nadkarni@lshtm.ac.uk

Organisational affiliation of the review

London School of Hygiene and Tropical Medicine
https://www.lshtm.ac.uk/

Review team members and their organisational affiliations

Dr Abhijit Nadkarni. London School of Hygiene and Tropical Medicine
Ms Yashi Gandhi. Sangath
Myrthe van den Broek. War Child Holland

Type and method of review

Intervention, Narrative synthesis, Systematic review

Anticipated or actual start date

04 May 2021

Anticipated completion date

04 July 2021

Funding sources/sponsors

• NIMH [Grant number: 1U01MH115504-01]

• Fund granted to War Child Holland by a foundation that wishes to remain anonymous

Grant number(s)

State the funder, grant or award number and the date of award

NIMH [Grant number: 1U01MH115504-01]

Conflicts of interest

Language
English

Country
England, India, Netherlands, United States of America

Stage of review
Review Ongoing

Subject index terms status
Subject indexing assigned by CRD

Subject index terms
Developing Countries; Humans; Income; Mental Health; Mental Health Services

Date of registration in PROSPERO
20 May 2021

Date of first submission
18 May 2021

Stage of review at time of this submission

Stage	Started	Completed
Preliminary searches	Yes	No
Piloting of the study selection process	No	No
Formal screening of search results against eligibility criteria	No	No
Data extraction	No	No
Risk of bias (quality) assessment	No	No
Data analysis	No	No

The record owner confirms that the information they have supplied for this submission is accurate and complete and they understand that deliberate provision of inaccurate information or omission of data may be construed as scientific misconduct.
The record owner confirms that they will update the status of the review when it is completed and will add publication details in due course.

Versions
20 May 2021
