# Supplementary material for: Interventions to increase help-seeking for mental health care in low- and middle-income countries: A systematic review
Source: PLOS Glob Public Health. 2023 Sep 13;3(9):e0002302. doi: 10.1371/journal.pgph.0002302 (PMC10499262; doi:10.1371/journal.pgph.0002302)
Supplement: Attachment — Submitted filename: Response to reviewers_PGPH-D-22-01985_12Jul23.docx [file pgph.0002302.s006.docx]

**Manuscript:** “Interventions to Increase Help-seeking for Mental Health Care in Low-and-Middle-Income Countries: A Systematic Review.” (PGPH-D-22-01985)

12^th^ July 2023

Dear Dr. Govindasamy,

Thank you for the detailed comments and for the invitation to revise and submit our manuscript to PLOS Global Public Health. We have revised the manuscript based on the comments and described the revisions below.

**Reviewer comments**

**Reviewer #1**

Abstract
Line 13: This treatment gap is particularly large in in low- and middle-income countries (LMIC) and is due to both supply-side and demand-side barriers.
Repetition of the word "in"

**Response:** Thank you for pointing it out. We have removed the additional “in”.

Introduction
The authors provide sufficient background to an important public health issue - mental health/illness, particularly in the LMIC context and highlight disparities between LMICs and HICs in addressing the problem. They outline the barriers to access and uptake of mental health services in LMICs and highlight the need for scalable innovations to reduce the treatment gap.

In their rationale for the SR, the authors describe the theory underpinning the majority of help-seeking interventions, and outline the contributions of previous work to the body of knowledge, which helps to highlight the gaps they intend to fill and importantly, they acknowledge the need for contextual strategies to improve help-seeking in LMICs.

Line 43: Many efforts have been made to overcome supply-side barriers….Can you name some of these supply side barriers to provide the reader more context?

**Response:** We have now added a couple of examples to this sentence in the Introduction section to address this: “Many efforts have been made to overcome supply-side barriers (e.g., lack of trained professionals, limited financial resources) to mental health care by increasing access to, and availability of, care aiming to reduce the treatment gap for mental disorders.”

The aims and objectives of the SR are clearly defined.

Methods
The search strategy was appropriate and the process rigorous. Eligibility criteria is more inclusive in comparison to previous reviews. However, the authors chose to include studies published in English only, which is noted later in the manuscript as a limitation of the study.

Line 94: concepts: ‘mental health/illness’ (e.g., anxiety, psychos?s).
Typo on spelling of psychosis.

**Response:** We have corrected this and now changed it to “psychosis”.

Results
The authors provide a comprehensive summary of the included studies and describe the various interventions which have been grouped into three intervention categories following the steps in the help-seeking process. A summary of how the interventions were implemented is presented and outlines the who, what and where. The effectiveness of the interventions is presented according to the intervention categories previously mentioned and various combinations of two or all of intervention categories. Implementation outcomes, feasibility (including barriers to implementation), appropriateness are also presented.

Discussion and Conclusion
The discussion is well aligned with the aim stated in the introduction which was to identify and synthesise the evidence on interventions to improve help-seeking for mental health problems in LMICs. The authors have summarised the key findings and interpreted the results appropriately. I would have liked to see some of the LMIC countries or continents of included studies mentioned in the discussion for context.

**Response**: Thank you for this suggestion. We have now included countries in the Discussion section.

The authors note the contributions of this important piece of work to this topic and acknowledge some important limitations of the study. Implications for future research are also noted. The final conclusions made are supported by the data.

The acronym ASHA for Accredited Social Health Activists is only written in full in the footnote of Table 1. Please write in full at first mention in the manuscript text (Line 551?).

**Response**: Thank you, we have added that as “Accredited Social Health Activists (ASHAs)” in the manuscript text in the Discussion section.

**Reviewer #2**

Article overview
In this study, the authors sort to identify interventions to improve help-seeking for mental health problems in LMICs. To investigate possible causes of the gap existing between mental health detection and linkage to care/treatment, the authors conducted a systematic review for evidence of interventions that actively sought to bridge this gap. Specifically, their synthesis focused on interventions aimed at improving help-seeking for mental health problems in LMICs. Although not framed in a formal question, the authors state that their review aimed to identify and synthesise the evidence on interventions to improve help-seeking for mental health problems in LMICs. The authors identified 42 articles that are relevant to the topic under investigation. They report that interventions using a combination of interventions to target health-seeking showed the most improvement in help-seeking behaviour and eventual linkage to mental health therapy/treatment. However, due to the limited number of robust studies done in LMICs and the heterogeneity of study designs, outcomes and activities used, the authors could not reach any definite conclusions regarding the effects of individual strategies or content of the interventions.

Reviewer comments to author
The article is well presented and easy to follow. It touches on a very important topic as most people in LMICs are either unaware of mental health problems (or what mental health entails) or are do not know that help is available. I do, however, have the following minor points that need clarification:

Line 151: The authors show that they conducted risk of bias analysis using JBI in supplemental documentation (S4). However, the scoring (or how subsequent decisions were made based on the JBI appraisal) is not evident (e.g. score ranges and weighting, were there any cut-offs suggested to guide decisions around bias, what was deemed acceptable vs not, etc.).

**Response**: Thank you for raising this concern. We have included the following line in the Methods section: “All assessed papers were included in the review, irrespective of the scoring or quality.” We also addressed this as a limitation in our Limitation section: “Furthermore, although the risk of bias was assessed, the quality of each study did not impact the weight given to it in the narrative synthesis.”

Lines 176-177
The authors state: “Half of the interventions targeted people experiencing mental health problems (n=18)”. Is this a reference to another total (i.e. n=36), because earlier it is reported that 42 eligible studies were identified (line 172). If so, then perhaps this sentence can be rephrased so as to prevent any confusion.

**Response**: Thank you for pointing this out. We have now corrected the statement in the Methods section to read as follows: “Nearly 40% of the interventions targeted people experiencing mental health problems (n=18), of which four focused on pregnant women. The other 60% focused on general population *and* people experiencing mental health problems, of which six focused on children, and adolescents.”

Lines 275-276
Minor comment. The numbering of “effects” is similar to reference citing in the manuscript. Perhaps using 1 bracket instead of 2 could avoid possible confusion [ i.e. 1) instead of (1)].

**Response**: We have made the suggested change in the Results section.

General comments on Tables and Figures
Figure 1: In screening, under “Full-text reports assessed for Eligibility”, it is suggested that the authors state the total number of articles before detailing how they went about assessing eligibility. Adding totals will maintain consistency with other sections in the diagram.

**Response**: We have made this change in Figure 1 and it now reads as: “Full-text reports assessed for Eligibility= 744” followed by the break down.

In addition, the authors might have explained how they moved from “Reports sought for retrieval=730” to “Number of included studies=42” in the body on the manuscript. However, this does not come across clearly in the diagram since 702 reports were excluded from the 730.

**Response**: Thank you for raising this concern. As suggested above, we have added a total next to “Full-text reports assessed for eligibility” which is 744 in Figure 1. Out of these, 702 were excluded and thus, “Number of studies included= 42”.

Tables 1-3: Although captions are clear enough for reader to understand what is being communicated, they, however, seem too general. It is suggested that authors be a bit more descriptive to enhance captions.

**Response**: Thank you for this suggestion, we have adapted the captions of all tables.

**Editor’s comments:**

Please review your reference list to ensure that it is complete and correct. If you have cited papers that have been retracted, please include the rationale for doing so in the manuscript text, or remove these references and replace them with relevant current references. Any changes to the reference list should be mentioned in the rebuttal letter that accompanies your revised manuscript. If you need to cite a retracted article, indicate the article’s retracted status in the References list and also include a citation and full reference for the retraction notice.

**Response:** Some in text citations were not correctly automated, but were already in the reference list and used in the manuscript elsewhere. The in text citations have been updated as needed.

Please provide separate figure files in .tif or .eps format only and remove any figures embedded in your manuscript file. Please also ensure all files are under our size limit of 10MB.

For more information about figure files please see our guidelines:

<https://journals.plos.org/globalpublichealth/s/figures> 
<https://journals.plos.org/globalpublichealth/s/figures#loc-file-requirement>

**Response:** We have now submitted the figure as .tif file.

We notice that your supplementary tables are uploaded with the file type 'Figure'. Please amend the file type to 'Supporting Information'. Please ensure that each Supporting Information file has a legend listed in the manuscript after the references list.

**Response:** Thank you, we have now made this change.

In the online submission form, you indicated that "The data that support the findings of this study are available from the corresponding author upon request". All PLOS journals now require all data underlying the findings described in their manuscript to be freely available to other researchers, either 1. In a public repository, 2. Within the manuscript itself, or 3. Uploaded as supplementary information. This policy applies to all data except where public deposition would breach compliance with the protocol approved by your research ethics board. If your data cannot be made publicly available for ethical or legal reasons (e.g., public availability would compromise patient privacy), please explain your reasons by return email and your exemption request will be escalated to the editor for approval. Your exemption request will be handled independently and will not hold up the peer review process, but will need to be resolved should your manuscript be accepted for publication. One of the Editorial team will then be in touch if there are any issues.

**Response:** Thank you for pointing this out. Our protocol and search strategy have already been uploaded as supplementary appendix. Along with that, our data extraction sheet [an excel file] which includes study characteristics, intervention and outcome-related data will be made available on the London School of Hygiene and Tropical Medicine’s repository.

We would also like to let you know that we have made a few additions in the manuscript in track changes. We thank you for the review and helpful feedback. We look forward to hearing from you.

Best wishes,

Abhijit Nadkarni

NIHR Professor of Global Research| Department of Population Health | London School of Hygiene & Tropical Medicine, United Kingdom

Co-Director | Addictions and related Research Group | Sangath, Goa, India
